# Information-Geometric Optimization with Natural Selection

**DOI:** 10.3390/e22090967

**Published:** 2020-08-31

**Authors:** Jakub Otwinowski, Colin H. LaMont, Armita Nourmohammad

**Affiliations:** 1 Max Planck Institute for Dynamics and Self-Organization, 37077 Göttingen, Germany; colin.lamont@ds.mpg.de (C.H.L.); armita@ds.mpg.de (A.N.); 2 Physics Department, University of Washington, Seattle, WA 98195, USA; 3 Fred Hutchinson Cancer Research Center, Seattle, WA 98109, USA

**Keywords:** optimization, population genetics, quantitative genetics

## Abstract

Evolutionary algorithms, inspired by natural evolution, aim to optimize difficult objective functions without computing derivatives. Here we detail the relationship between classical population genetics of quantitative traits and evolutionary optimization, and formulate a new evolutionary algorithm. Optimization of a continuous objective function is analogous to searching for high fitness phenotypes on a fitness landscape. We describe how natural selection moves a population along the non-Euclidean gradient that is induced by the population on the fitness landscape (the natural gradient). We show how selection is related to Newton’s method in optimization under quadratic fitness landscapes, and how selection increases fitness at the cost of reducing diversity. We describe the generation of new phenotypes and introduce an operator that recombines the whole population to generate variants. Finally, we introduce a proof-of-principle algorithm that combines natural selection, our recombination operator, and an adaptive method to increase selection and find the optimum. The algorithm is extremely simple in implementation; it has no matrix inversion or factorization, does not require storing a covariance matrix, and may form the basis of more general model-based optimization algorithms with natural gradient updates.

## 1. Introduction

Finding the optimal parameters of a high dimensional function is a common problem in many fields. We seek protein conformations with minimal free energy in biophysics, the genotypes with maximal fitness in evolution, the parameters of maximum likelihood in statistical inference, and optimal design parameters in countless engineering problems. Optimization can be visualized as climbing a landscape representing the objective function along the steepest directions. Often derivatives of the objective function are not available or are too costly, and derivative-free algorithms must be applied.

Evolutionary algorithms aim to find the optimum of an objective “fitness” function, f(x) with parameters *x* representing genetic or phenotypic variants. Algorithms differ in how new variants are generated and how the set of variants (the population) shifts over the course of the algorithm (selection). A class of algorithms called genetic algorithms are most directly inspired by the Wright–Fisher and Moran models from population genetics [1,2]. Genetic algorithms use mutation and recombination operators to generate new variants, and some form of biased stochastic reproduction to select variants for the next generation. Among the different stochastic selection schemes, fitness-proportionate selection is equivalent to natural selection in population genetics [1].

Stochasticity of reproduction, known as genetic drift in population genetics, is a natural phenomenon present in finite populations and has the important effect of scaling selection inversely with the magnitude of stochasticity (i.e., population size) [2]. Stochastic selection is very inefficient in that it causes the loss of many genotypes. For example, in the regime of strong selection and weak (i.e., rare) mutation of the Moran model, the probability that a single genotype will sweep a population (fixation) is proportional to its selective advantage. For a variant with fitness advantage of 1%, typical in bacteria, there is a 99% chance it will go extinct [2].

In evolutionary optimization, stochasticity of reproduction may be helpful in exploring noisy or rugged fitness functions, but it is not essential if there are other sources of stochasticity. Some genetic algorithms use deterministic rank-based selection which removes individuals below some threshold [1]. However, such truncation selection is very coarse, and does not affect proportionally the variants that survive.

Another class of evolutionary algorithms, which includes estimation of distribution algorithms [3] and algorithms known as evolution strategies [4,5], are based on defining a population as a parameterized distribution P(x|θ). These algorithms differ from genetic algorithms in that there are no selection or mutation operators applied directly to individuals in a population. In these algorithms, variants are generated by drawing from the distribution, and selection amounts to updating the parameters θ in the direction of higher population mean fitness, i.e., the gradient $\nabla_{\theta }F$. To account for the uncertainty of the parameters some algorithms move the parameters in the direction of the natural gradient, $g^{-1}\nabla_{\theta }F$, where $g^{-1}$ is the inverse of the Fisher information which can be estimated from the population [6]. The popular covariance matrix adaptation evolutionary strategy (CMA-ES) [7,8] and closely related natural evolution strategies (NES) [9,10,11,12] parameterize a population as a normal distribution, and use variants drawn from the distribution to update the mean and covariance with a natural gradient descent step. More generally, natural gradients describe the ascent of the fitness function in terms of information geometry, and their use characterizes a wide class of information-geometric algorithms [13]. Computing the natural gradient in these algorithms often requires matrix factorization or inversion, and explicitly calculating parameters, such as the covariance matrix, can be costly if the dimensionality is high.

Here, we develop an evolutionary algorithm, similar to CMA-ES/NES, that uses deterministic natural selection to incorporate the natural gradient, without a need to store a covariance matrix or any costly linear algebra operations. We show that the natural gradient used in information-geometric optimization is also a fundamental part of natural selection. We show how natural selection is related to Newton’s method in optimization on a quadratic fitness landscape. We describe how the choice of the scale of selection has to balance between biasing towards high fitness and losing information of the underlying fitness function. We show how to generate new variants with a new recombination operator, without having to explicitly estimate a covariance matrix. Finally, we develop a proof-of-principle quantitative genetic algorithm (QGA) which iterates between generating variants with our recombination operator and a method of tuning the scale of selection to converge to an optimum.

## 2. Natural Selection Gradient

We begin by considering a population of infinite size and with a finite number of unique variants called phenotypes. A variant *i* is defined by a continuous multivariate phenotype $x_{i}$ (a vector), a frequency $p_{i}$ and growth rate, or fitness $f(x_{i})$. Note that a variant does not represent an individual organism, but a number of organisms with the same phenotype. In the context of optimization, phenotypes are candidate solutions, and fitness is the objective function to be maximized. Classical replicator dynamics, leaving out mutation and genetic drift, describes the change in frequencies due to selection as

(1) $$\frac{dp_{i}}{dt}=p_{i}\left(f(x_{i})-F\right),$$

with means fitness $F=\sum_{i}p_{i}f\left(x_{i}\right)$. In stochastic descriptions, these dynamics describe the expected change due to selection.

**Figure 1:**
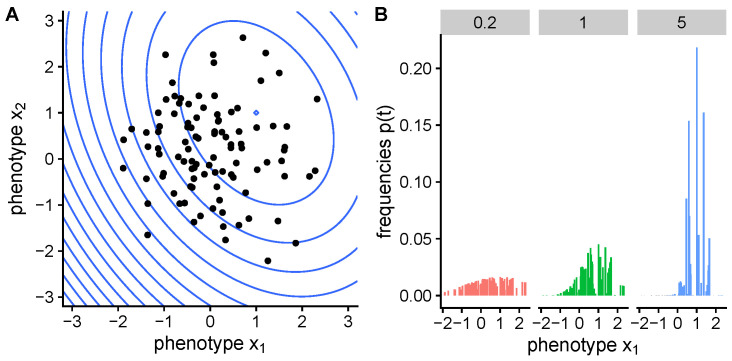
(**A**) An example of 100 variants in a 2D phenotype space on a quadratic fitness landscape (blue contours). (**B**) Frequencies evolve over time according to Equation (2), with t=0.2 (red), t=1 (green) and t=5 (blue).

In the absence of other processes, frequencies can be integrated over time resulting in

(2) $$p_{i}\left(t\right)=p_{i}\left(0\right)\frac{1}{Z_{t}}e^{tf(x_{i})},$$

with normalization $Z_{t}=\sum_{i}p_{i}\left(0\right)e^{tf(x_{i})}$. At long time *t*, the phenotype distribution will concentrate on high fitness phenotypes until the highest fitness phenotype reaches a frequency of unity. The change in mean fitness equals the fitness variance (Fisher’s theorem [14]), and higher moments evolve as well. As an example we show 100 variants in a quadratic fitness landscape and how their frequencies change over time (Figure 1).

Remarkably, replicator dynamics can be rewritten in terms of information geometry [15]. The distribution over phenotypes can be described as a discrete categorical distribution, with parameters p, the vector of (linearly independent) frequencies. Natural selection adjusts these parameters in the direction of the covariant derivative [16,17], also known as the natural gradient [6],

$$\frac{dp}{dt}=g^{-1}\nabla_{p}F,$$

where $\nabla_{p}F$ is the vector of partial derivatives of the mean fitness, and $g^{-1}$ is the inverse of the Fisher information metric of the categorical distribution. This metric defines distances on the curved manifold of probability distributions (see Appendix A). Selection changes the frequencies in the direction of steepest ascent in non-Euclidean coordinates defined by the geometry of the manifold of the distribution.

The natural gradient is a geometric quantity independent of parameterization. Therefore, if the distribution over x can be well approximated by another distribution, selection will also change those parameters in the direction of the natural gradient. This can be demonstrated by projecting onto a normal phenotype distribution, as is assumed in classic multivariate quantitative genetics. The population mean μ and population covariance matrix Σ parameterize the distribution, and selection changes the mean as ([18,19], Appendix A)

(3) $$\frac{d\mu }{dt}=\Sigma \nabla_{\mu }F,$$

where $\Sigma^{-1}$ is the associated Fisher information metric. Similarly, the covariance follows a natural gradient with a more complex metric (Appendix A). If phenotype covariance reaches zero, then the population is monomorphic and there is zero selection. However, a related population genetics model in the limit of small diversity can search a fitness landscape with the help of mutations, with a covariance matrix defined by mutations serving as a metric (Appendix E).

For a finite amount of time, the frequencies have Boltzmann form and the parameters trace a path on the manifold of distributions following the natural gradient. Exponential weights lead to natural gradient updates and are found in many optimization algorithms beyond GAs, such as simulated and population annealing [20,21]. In contrast, CMA-ES/NES uses a population to update the parameters of a normal distribution in a natural gradient step, and does not track frequencies.

## 3. Natural Selection Uses Second Derivatives

Natural gradient descent uses second derivatives, and is equivalent to Newton’s method on a quadratic objective function [6]. Analogously, natural selection on a population of finite variants is related to Newton’s method on a quadratic fitness landscape.

Phenotypes remain normally distributed under replicator dynamics on a quadratic fitness landscape, which can be seen by using Gaussian frequencies in Equation (2). The mean and covariance change as (Appendix B)

(4) $$\begin{array}{cc} \mu_{t}-\mu_{0}\; & =t\Sigma_{0}\nabla f\\ \Sigma_{t}^{-1}-\Sigma_{0}^{-1}\; & =tC \end{array}$$

where subscripts indicate dependence on time; ∇f is the fitness gradient, evaluated at $\mu_{0}$; C is the fitness curvature—that is, the negative of the matrix of second derivatives of fitness, evaluated at $\mu_{0}$. The change in mean is a finite natural gradient step, while the covariance aligns itself with the curvature over time. Combining the two equations yields

(5) $$\mu_{t}-\mu_{0}={\left(\frac{1}{t}\Sigma_{0}^{-1}+C\right)}^{-1}\nabla f.$$

In the asymptotic regime of large time *t*, $\mu_{t}-\mu_{0}\to C{}^{-1}\nabla f$ corresponding to an iteration of Newton’s method. However, at large time *t* the distribution is no longer normally distributed due to the finite number of variants (see below). For small and intermediate time *t*, selection behaves as a form of regularized Newton’s method, where *t* determines how much to weigh the initial distribution. Since $\Sigma_{t}$ is always positive-definite, the population should not converge to a saddle point in fitness, which is possible in Newton’s method.

## 4. Selection Reduces Diversity

**Figure 2:**
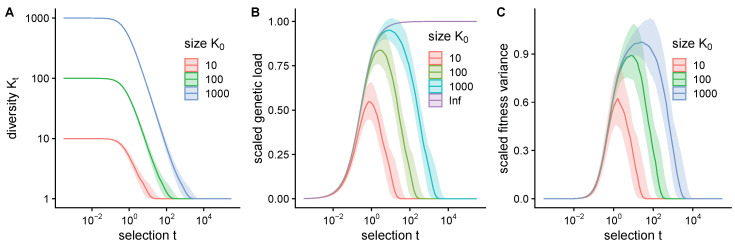
Selection reduces diversity and at higher levels of selection the population is no longer normally distributed. Fitness landscape is shown in Figure 1A. (**A**) The effective number of variants, $K_{t}$, shrinks with increased selection and depends on the total number of variants (colors). Populations were drawn 1000 times from a standard normal with solid lines indicating the median value and shaded regions indicating the middle 50% of the distribution. (**B**) Scaled genetic load $t(F^{†}-F)$, that is, the gap between the maximum observed fitness $F^{†}$ and mean fitness *F*, has a peak value at intermediate selection due to the loss of diversity at strong selection. Purple line indicates continuous limit of a normal distribution (Appendix C). (**C**) Fitness variance (multiplied by $t^{2}$ to make it unit-less) also peaks at some intermediate level of selection.

Natural selection moves the distribution along a manifold shaped by the fitness landscape. However, selection does not introduce any new phenotypes, and reduces diversity by biasing frequencies towards high fitness phenotypes. Diversity can be quantified by population entropy $S_{t}=-\sum_{i}p_{i}\left(t\right)\log p_{i}\left(t\right)$, which summarizes the variation in the distribution of frequencies. The exponential of entropy $K_{t}=e^{S_{t}}$ defines an effective number of variants, such that $1\leq K_{t}\leq K_{0}$, and $K_{0}$ is the number unique variants under uniform initial conditions $p_{i}\left(0\right)=1/K_{0}$. Diversity shrinks rapidly at large time *t*, depending on the starting point $K_{0}$ (Figure 2A).

Furthermore, for large time *t* and small diverity $K_{t}$ the approximation of normally distributed phenotypes no longer holds, and Equation (5) does not hold even if fitness is purely quadratic. The breakdown of normality is reflected in the moments of the fitness distribution. The gap between the mean and maximum possible fitness $F^{*}-F$, known as genetic load, shrinks as time increases, but under normal phenotype distributions, $F^{*}-F\to \frac{D}{2t}$ where *D* is the dimensionality of phenotypes (Appendix C). Fitness has units of $t^{-1}$, as evident from Equations (1) and (2). The unit-less approximate scaled load $t(F^{†}-F)$ where $F^{†}$ is the maximum observed fitness, is zero when mean fitness approaches the maximum, and reaches a peak around some intermediate level of selection (Figure 2B). Similarly, the fitness variance scaled by $t^{2}$ has a peak at intermediate selection (Figure 2C) since fitness variance must be zero at high selection.

We can regard time *t* as the scale of selection, as it directly multiplies the fitness. There is an inherent tradeoff in choosing the scale of selection, in that weak selection weakly biases the frequencies towards high fitness, and strong selection has low diversity, and the population is not well approximated by a normal distribution.

## 5. Generating New Variants

As selection *t* increases, the population of phenotypes x moves up the natural gradient (Equation (2)). To make an effective evolutionary optimization algorithm, we need to generate new variants based on the existing parent population.

A population with large enough diversity on a quadratic fitness landscape is approximately normally distributed, and the weighted population mean μ and weighted population covariance matrix Σ can be interpreted as the weighted maximum likelihood estimates of the mean and covariance. Therefore, new variants can be generated by estimating these parameters and drawing from the distribution, as in CMA-ES/NES. In contrast to those algorithms, we do not need to explicitly calculate a natural gradient step, since this is done by tuning the weights of the variants. Instead of pursuing this approach, we draw more inspiration from biology.

## 6. Recombination Efficiently Generates Diversity

While selection moves the mean phenotype toward an optimum at the cost of reducing diversity, diversity can be restored by mutation and recombination processes that add new variants to the population. Biologically, mutations and recombination are changes in genotypes and their effect on phenotypes depends on the genotype-phenotype map. Mutations generate new variants that, if beneficial, may rise to some frequency after selection, and the expected cost or benefit of a mutation depends on the fitness landscape.

As an alternative to specifying a genotype-phenotype map, we define a mutation as a small stochastic normally distributed change in phenotype space. Whether new variants have higher or lower fitness than the exisiting population depends on the fitness landscape. Generally, fitness landscapes will have some directions which are very steep and some that are shallow. If a population is on such a high dimensional ridge, where most directions are costly, then a mutation is very likely to be very costly. For normally distributed populations and mutations, the expected fitness cost is proportional to the effective number of directions which are deleterious (Appendix D).

From an optimization perspective, mutations are very inefficient in that they rarely generate good solutions since they are poorly aligned with the fitness landscape. However, recombination has the remarkable property of being adaptive to the fitness landscape. In population genetics models with genotypes, recombination is known to quickly break down correlations between sites in a genome until linkage equilibrium is reached [22,23]. Under these conditions, the phenotype covariance and expected fitness of recombined offspring is the same as the parent population [22,23]. If the population is on a high dimensional ridge and the population covariance is aligned, recombined solutions would also be aligned with the ridge. However, linkage equilibrium can only be reached on fitness landscapes without interactions between genetic sites, which excludes most fitness landscapes, including quadratic fitness-phenotype maps.

For the purposes of optimization, we are free to define any recombination operator. Genetic algorithms typically use recombination in the form of a crossover operator, similar to genetic recombination. However, a naive application of crossover to phenotypes would destroy any covariance in the population. Here, we define recombination of phenotypes that preserves covariance. A pair of distinct phenotypes, chosen by weighted random sampling with weights $p_{1}\left(t\right)$ and $p_{2}\left(t\right)$ can be recombined as

(6) $$x^{\prime }=\mu_{t}+\frac{1}{\sqrt{2}}\left(x_{1}-x_{2}\right),$$

which has the same expectation $\mu_{t}$ and covariance $\Sigma_{t}$ as the parent population. This recombination operator preserves normal statistics, and is a way of sampling from an implicit normal distribution without estimating its parameters. This operator resembles the mutation operator in differential evolution optimization, which also preserves normal statistics for certain parameter values [24]. However, the error in the mean and covariance can be large when diversity is low due to the finite number of variants, and the number of unique recombinants can be too limited. We improve the quality of recombination by generalizing to a stochastic sum over the entire population

(7) $$x^{\prime }=\mu_{t}+\sum_{i}\eta_{i}\sqrt{p_{i}\left(t\right)}\left(x_{i}-\mu_{t}\right),$$

where $\eta_{i}$ is a random variate drawn from a standard normal random distribution N(0,1). This operator also conserves normal statistics, tuned by the scale of selection *t*, and efficiently generates new variants without storing a covariance matrix. In comparison, CMA-ES/NES must store a covariance matrix, which may be challenging in large problems.

## 7. Quantitative Genetic Algorithm

We define a proof-of-principle quantitative genetic algorithm (QGA), as it is related to quantitative genetics, and is also a genetic algorithm that is quantitative in the sense that it tracks frequencies of types. QGA iterates between tuning the scale of selection and generating new variants.

A critical problem is how to increase, or sometimes decrease, selection over the course of optimization to make sure the population ends extremely closely distributed around the optimum. In QGA, variants are given Boltzmann weights (Equation (2)), with $p_{i}\left(0\right)=1/K_{0}$, and the choice of the scale of selection *t* determines how much the population moves up the natural gradient at the cost of losing diversity. One choice is a fixed schedule of increases in selection *t*, as in simulated annealing and population annealing [20,21]. However, choosing a good rate is challenging as increasing selection too quickly will lead to premature convergence, and increasing selection too slowly wastes computational resources. In addition, a constant rate may not work well when different parts of the fitness landscape have different curvatures.

It is clear that some intermediate level of selection is needed, although the peak in scaled genetic load or fitness variance is not necessarily the right balance. We implement an adaptive strategy that keeps diversity fixed and lets selection vary on its own. After a round of generation, population entropy will typically increase by a small amount. Then we choose a new scale of selection *t* such that the entropy is at a target value *S*, which is a hyper-parameter of the algorithm. This way the population adapts to keep the diversity constant, with high fitness variants driving selection higher as they are found. If the diversity *S* is too small the algorithm can still converge prematurely outside of the local quadratic optimum—i.e., on a slope.

For generating variants, we use the recombination operator Equation (7), which has the same expected mean and covariance as the parent population. The most common approach is to operate in generations, where the population is completely replaced each round, at the cost of losing information from previous generations. Here, we take a different approach that discards information only when it becomes irrelevant. We generate only one variant per iteration, and integrate it into the existing population. The parent population has Boltzmann weights and the ensemble of possible recombinants are unweighted, and there is ambiguity in how to combine these different statistical ensembles. We found that the simplest approach is the most practical, which is to add each new variant to the population, and in the next selection step recalculate the Boltzmann weights. We can further simplify the implementation, by fixing the number of variants, discarding the variant with the smallest weight and replacing it with the new generated variant.

Furthermore, we modify the recombination operator by replacing the mean $\mu_{t}$ in Equation (7) with the variant with the highest fitness observed thus far, equal to the mean at infinite selection $\mu_{\infty }$. Shifting the mean of the recombinant distribution was found to perform much better in benchmarks (see below). The resulting algorithm (Algorithm 1) is very simple compared to CMA-ES, since it requires no matrix inversion or decomposition, and the only hyper-parameters are the target entropy *S* and how the initial population of variants is generated. We provide an implementation online, with minor implementation details described in Methods.
**Algorithm 1** Quantitative genetic algorithm.
1: Choose hyperparameter *S*2: Draw population $x_{i}$ for all *i* from initial normal distribution3: **repeat**4:  Find *t* such that $S=-\sum_{i}p_{i}\left(t\right)\log p_{i}\left(t\right)$5:  Sample standard normal variates $\eta_{i}$ for all *i*6:  Add to population $x^{\prime }\leftarrow \sum_{i}\eta_{i}\sqrt{p_{i}\left(t\right)}\left(x_{i}-\mu_{\infty }\right)+\mu_{\infty }$7: **until** convergence criteria met


## 8. QGA Performance

**Figure 3:**
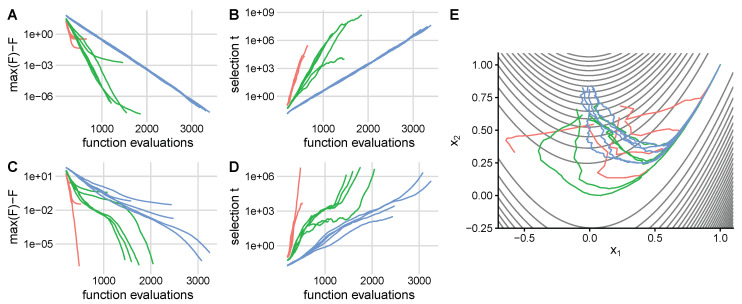
QGA converges to the optimum when there is enough diversity. Optimization on an ellipsoid (**A**,**B**) and a non-convex Rosenbrock function (**C**–**E**) (see Methods) was carried out for three different values of entropy *S*: 3 (green), 4 (red), and 5 (blue), and 5 independent runs each. For the ellipsoid, distance to maximum fitness decreases exponentially (**A**) and selection increases exponentially (**B**), unless the population converges prematurely. For the Rosenbrock function, the approach to the optimum fitness (**C**) and increase in selection (**D**) are non-exponential as selection is adapted to keep the population entropy *S* at target values. (**E**) Paths in phenotype space to the optimum.

First, we demonstrate QGA on two simple test functions (see Methods). Optimization of a 5-dimensional quadratic function (Figure 3A,B) converges to the optimum for large target entropy *S*, and converges outside of the optimum for smaller entropy. The selection scale *t* increases exponentially with the number of evaluations when the algorithm is converging. On a non-convex 2-dimensional Rosenbrock function (Figure 3C–E) selection is tuned according to the curvature of the landscape over the course of the optimization. Initially, selection increases rapidly as the population falls into the main valley, then selection slows down as it moves along the flatter part of the valley floor, and finally speeds up again as it converges to a quadratic optimum.

**Figure 4:**
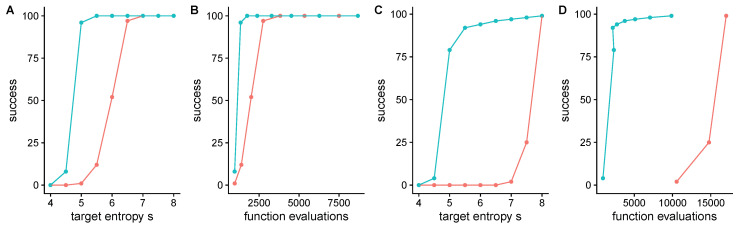
QGA performance on 5-dimensional quadratic (**A**,**B**) and Rosenbrock (**C**,**D**) functions from the blackbox optimization benchmark library with different values of target diversity *S*. The best choice of *S* maximizes the chance of convergence and minimizes the number of function evaluations. (**A**,**C**) Successful convergence as a function of *S* in 100 instances with randomized optima, where success is when the lowest value found is within $10^{-8}$ of the true minimum after less than $5\times 10^{4}$ evaluations. (**B**,**D**) Success as function of the median number of function evaluations conditional on success, with *S* as in (**A**,**C**). Red lines indicate standard recombination, Equation (7), and blue lines indicate modified recombination (see text). Each population was initialized with mean zero and standard deviation of 3 in each dimension.

To more rigorously assess performance, we tested QGA on noiseless test functions from the blackbox optimization benchmark library [25] (implemented in [26]), which implement an ensemble of “instances” of each function with randomized locations of optima. On 5-dimensional quadratic and Rosenbrock functions, the chance of convergence to the optimum, and the average number of function evaluations is sensitive to the hyper-parameter target entropy *S*, with low *S* leading to premature convergence, and high *S* leading to a high number of function evaluations (Figure 4 red lines). Performance is significantly improved with the modified recombination operator which replaces $\mu_{t}$ in Equation (7) with the best variant $\mu_{\infty }$. This modified algorithm is able to make larger steps towards the optimum with lower values of *S* without prematurely converging (Figure 4 blue lines).

**Figure 5:**
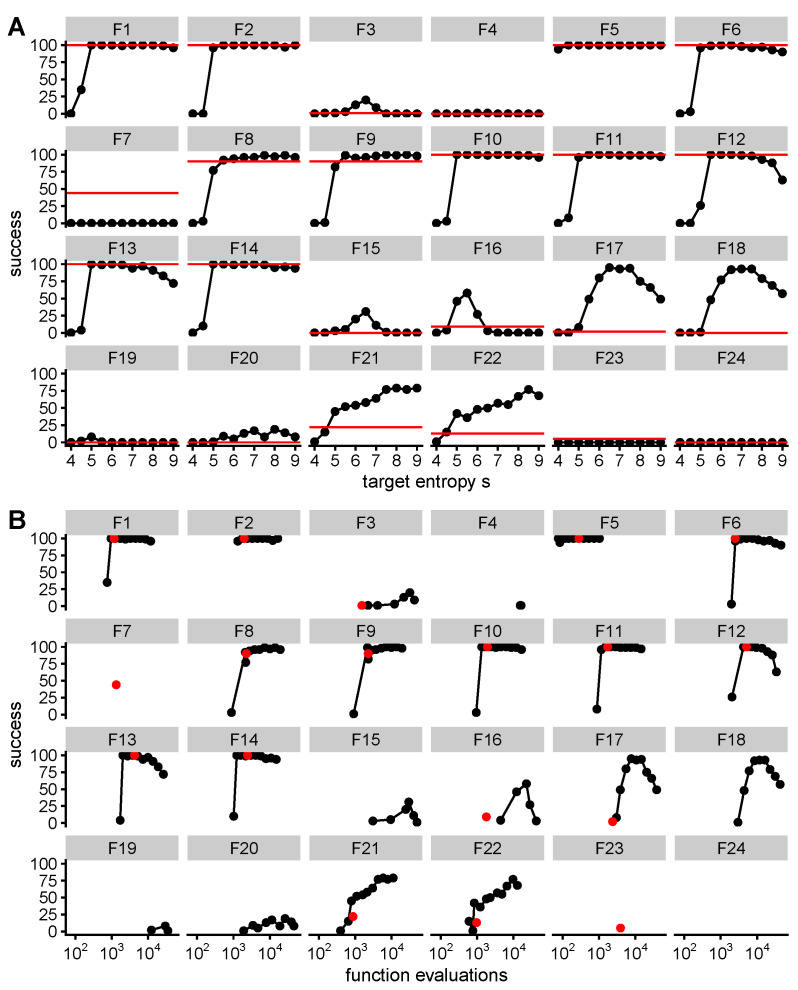
QGA performance is similar to CMA-ES on 24 noiseless functions from the blackbox optimization benchmark library with 5 dimensions and different values of target entropy *S*, with QGA (black) and CMA-ES (red). The best choice of *S* maximizes the chance of convergence and minimizes the number of function evaluations. (**A**) Successful convergence as a function of *S* in 100 instances with randomized optima, where success is when the lowest value found is within $10^{-8}$ of the true minimum after less than $5\times 10^{4}$ evaluations. (**B**) Success as a function of the median number of function evaluations conditional on success, with *S* as in A. Both algorithms had initial conditions of mean zero and three standard deviations in each dimension. F9 and F10 are the familiar Rosenbrock and ellipsoid functions respectively.

We tested QGA on all 24 noiseless test functions with the modified recombination operator over many values of target entropy *S* and compare to CMA-ES [26] (Figure 5). Overall, the performance of QGA with a good choice of *S* is very similar to CMA-ES, which may be expected due to their fundamental similarities. Both algorithms perform well on functions with strong global structure, where quadratic approximations may work well, and perform worse on functions with highly multimodal structure. QGA has higher chances of convergence for some of the multimodal functions (F15–F22). For the step ellipsoidal function (F7), QGA fails completely due to a check for numerical error (see Methods), although CMA-ES also performs poorly.

## 9. Discussion

We have shown how a fundamental object known by different names, the Boltzmann distribution in statistical physics, the softargmax function in computer science, and the replicator equation in evolutionary biology, performs natural gradient updates. We used these insights of the natural gradient, along with insights drawn from biological recombination, to create a novel evolutionary optimization algorithm that is remarkably simple and efficient.

In benchmarks, QGA requires more fine tuning of its hyper-parameter than CMA-ES. However, QGA is much simpler conceptually and in implementation compared to CMA-ES and NES. Those algorithms are somewhat complex and involve matrix inversions or decompositions, as well as adaptive learning rate schemes. QGA does not require any advanced linear algebra and does not store a covariance matrix explicitly, which may make it possible to use on higher dimensional problems, where storage of a covariance matrix may be an issue. Additionally, QGA naturally incorporates information from the history of the optimization, whereas CMA-ES/NES has “generations” of samples and incorporates past information more heuristically.

In comparison to the general class of genetic algorithms, QGA has deterministic selection which is much more efficient than stochastic fitness proportional selection. In addition, the recombination operator preserves relevant correlations between dimensions, in contrast to typical crossover operators.

We have shown that selection steps in evolutionary optimization algorithms and population genetics are intimately related, and we developed a way to control the increase in selection. Our method of increasing selection by fixing the population entropy is simple and adaptive, yet its implications are not entirely clear, and there may be other strategies that tune selection more efficiently. The challenge is to find observables that indicate premature convergence early enough to be able to continually adapt the diversity of the population.

Our recombination operator is efficient in that it obviates the need to calculate the covariance matrix. Our algorithm (and CMA/NES) model the population as normally distributed, so it may be useful to extend our scheme to other distributions. Since the natural gradient is independent of parameterization, it is possible to generalize our scheme to any distribution. Specifically, the parameters of a generative model can be estimated by maximum likelihood, with variants as data points with Boltzmann weights. This distribution is then used to generate new variants for the next iteration. The benefit of using our form of natural selection is that it is not an explicit natural gradient update, which requires second derivatives of the generative model. For natural selection, the limitation on diversity still applies, i.e., when selection is high, there are fewer degrees of freedom to approximate the objective function. Such an algorithm may be useful in generating high fitness variants for difficult problems, including complicated discrete spaces such as in the directed evolution of proteins.

## 10. Methods

### 10.1. Algorithm Details

Inputs are the chosen target entropy *S* (in base 2), and the mean and variance of the initial normal distribution from which $K_{0}=2^{S+1}$ variants are drawn. In the selection step, we calculate $p_{i}\left(t\right)=e^{tf(x_{i})}/Z_{t}$, where $Z_{t}=\sum e^{tf(x_{i})}$ and *t* is incremented or decremented geometrically by a small value until the entropy matches *S*. In the recombination step, a new variant is generated with an unbiased version of Equation (7), where $p_{i}\left(t\right)\to \frac{p_{i}\left(t\right)}{1-\sum_{i}e^{2tf(x_{i})}}$. To simplify the implementation, the list of variants is held at a fixed size $K_{0}$ by replacing the variant with the lowest probability with the new recombinant. Selection and recombination are iterated until the desired convergence criteria are met.

In addition, the algorithm is stopped when duplicate values of fitness for different parameters are detected in the population. The reason for this check is that often when the algorithm is prematurely converging, selection *t* diverges, and numerical error results in non-unique values of fitness. This check for numerical error causes the algorithm to fail on the step ellipsoidal function (F7).

Code available at github.com/jotwin/qga.

### 10.2. Test Functions

The test ellipsoid is $f\left(x\right)={\left(x_{1}+2x_{2}+3x_{3}+4x_{4}+5x_{5}\right)}^{2}$, and populations were initialized with 200 random variants drawn from a normal distribution with mean and variance equal to all one, so that populations are not centered on the optimum at zero. The test Rosenbrock function is $f\left(x\right)={\left(1-x_{1}\right)}^{2}+100{\left(x_{2}-x_{1}^{2}\right)}^{2}$, and populations were initialized with 200 random variants drawn from a normal distribution with mean (0,1) and standard deviation (0.25, 0.25).

## Appendix A. Natural Selection Gradient

We begin by following the appendix of [17] (see also [19]). A population has i=1…K variants with frequencies $p_{i}$ that evolve according to

(A1) $$\frac{dp_{i}}{dt}=p_{i}\left(f\left(x_{i}\right)-F\right),$$

with fitness *f*, phenotype $x_{i}$, and mean fitness $F=\sum_{i=1}^{K}p_{i}f\left(x_{i}\right)$. There are K−1 independent variables, so we choose the *K*th variant as the reference and

(A2) $$p_{K}=1-\sum_{i=1}^{K-1}p_{i}.$$

Eliminating this variable results in

(A3) $$\frac{dp_{i}}{dt}=\sum_{j=1}^{K-1}g_{ij}^{-1}\frac{\partial F}{\partial p_{j}},$$

with

(A4) $$g_{ij}^{-1}=p_{i}\delta_{ij}-p_{i}p_{j},$$

where $\delta_{ij}$ is the kronecker delta. $g_{ij}^{-1}$ is also the covariance of a categorical distribution. Note that indices are over the K−1 variables.

The inverse of this matrix is the Fisher information metric

(A5) $$\begin{array}{ccc} g_{ij} & = & \sum_{k=1}^{K}p_{k}\left(\frac{\partial }{\partial p_{i}}\log p_{k}\right)\left(\frac{\partial }{\partial p_{j}}\log p_{k}\right) \end{array}$$

(A6) $$\begin{array}{ccc} & = & \sum_{k=1}^{K}\frac{1}{p_{k}}\frac{\partial p_{k}}{\partial p_{i}}\frac{\partial p_{k}}{\partial p_{j}} \end{array}$$

(A7) $$\begin{array}{ccc} & = & \frac{1}{p_{K}}+\frac{1}{p_{i}}\delta_{ij} \end{array}$$

Each frequency $p_{i}$ has an associated phenotype $x_{i}$ (a vector of dimension D). The population mean and covariance of the phenotypes are

(A8) $$\begin{array}{ccc} \mu & = & \sum_{i=1}^{K-1}p_{i}\left(x_{i}-x_{K}\right)+x_{K} \end{array}$$

(A9) $$\begin{array}{ccc} \Sigma & = & \sum_{i=1}^{K-1}p_{i}\left[{\left(x_{i}-\mu \right)}^{2}-{\left(x_{K}-\mu \right)}^{2}\right]+{\left(x_{K}-\mu \right)}^{2} \end{array}$$

with shorthand for the matrix $b^{2}=bb^{⊤}$ given a vector b. Let us assume that the population is very diverse (*K* is very large) and it is well approximated by a normal distribution. In that case, the population mean μ and covariance Σ can be treated as parameters that fully characterize the population. The derivatives of mean fitness can be decomposed by the chain rule

(A10) $$\begin{array}{ccc} \frac{\partial F}{\partial p_{j}} & = & \frac{\partial \mu^{⊤}}{\partial p_{j}}\nabla_{\mu }F+\frac{\partial \Sigma^{⊤}}{\partial p_{j}}\nabla_{\Sigma }F \end{array}$$

(A11) $$\begin{array}{ccc} \frac{\partial \mu }{\partial p_{j}} & = & x_{j}-x_{K} \end{array}$$

(A12) $$\begin{array}{ccc} \frac{\partial \Sigma }{\partial p_{j}} & = & {\left(x_{j}-\mu \right)}^{2}-{\left(x_{K}-\mu \right)}^{2} \end{array}$$

where $\nabla_{b}$ is the vector of partial derivatives with respect to parameter b. The change in mean can be found by taking the time derivative of (A8), and using (A3), (A4), (A10)–(A12):

(A13) $$\begin{array}{ccc} \frac{d\mu }{dt} & = & \sum_{i=1}^{K-1}\left(x_{i}-x_{K}\right)\frac{dp_{i}}{dt} \end{array}$$

(A14) $$\begin{array}{ccc} & = & \sum_{i=1}^{K-1}\left(x_{i}-x_{K}\right)\sum_{j=1}^{K-1}g_{ij}^{-1}\frac{\partial F}{\partial p_{j}} \end{array}$$

(A15) $$\begin{array}{ccc} & = & \sum_{i=1}^{K-1}\left(x_{i}-x_{K}\right)p_{i}\left(\frac{\partial \mu^{⊤}}{\partial p_{i}}\nabla_{\mu }F+\frac{\partial \Sigma^{⊤}}{\partial p_{i}}\nabla_{\Sigma }F\right) \end{array}$$

(A16) $$\begin{array}{ccc} & & -\sum_{i=1}^{K-1}\left(x_{i}-x_{K}\right)p_{i}\sum_{j=1}^{K-1}p_{j}\left(\frac{\partial \mu^{⊤}}{\partial p_{j}}\nabla_{\mu }F+\frac{\partial \Sigma^{⊤}}{\partial p_{j}}\nabla_{\Sigma }F\right) \end{array}$$

(A17) $$\begin{array}{ccc} & = & \sum_{i=1}^{K-1}\left(x_{i}-x_{K}\right)p_{i}\left({\left(x_{i}-x_{K}\right)}^{⊤}\nabla_{\mu }F+{\left({\left(x_{i}-\mu \right)}^{2}-{\left(x_{K}-\mu \right)}^{2}\right)}^{⊤}\nabla_{\Sigma }F\right) \end{array}$$

(A18) $$\begin{array}{ccc} & & -\left(\mu -x_{K}\right)\sum_{j=1}^{K-1}p_{j}\left({\left(x_{j}-x_{K}\right)}^{⊤}\nabla_{\mu }F+{\left({\left(x_{j}-\mu \right)}^{2}-{\left(x_{K}-\mu \right)}^{2}\right)}^{⊤}\nabla_{\Sigma }F\right). \end{array}$$

All of the sums can be extended to *K* since the *K*th term is zero in each case, and this allows us to treat the sums as expectations over the variables. In addition we apply the identity $x_{i}-x_{K}=x_{i}-\mu +\mu -x_{K}$ to get

(A19) $$\begin{array}{ccc} \frac{d\mu }{dt} & = & \left(\Sigma +{\left(\mu -x_{K}\right)}^{2}\right)\nabla_{\mu }F+\left(\langle {\left(x_{i}-\mu \right)}^{3}\rangle +\Sigma \left(\mu -x_{K}\right)+{\left(x_{K}-\mu \right)}^{3}\right)\nabla_{\Sigma }F \end{array}$$

(A20) $$\begin{array}{ccc} & & -{\left(\mu -x_{K}\right)}^{2}\nabla_{\mu }F-\left(\Sigma \left(\mu -x_{K}\right)+{\left(x_{K}-\mu \right)}^{3}\right)\nabla_{\Sigma }F \end{array}$$

(A21) $$\begin{array}{ccc} & = & \Sigma \nabla_{\mu }F, \end{array}$$

where the third central moment $\langle {\left(x_{i}-\mu \right)}^{3}\rangle$ is zero for a normal distribution.

The change in covariance can be found similarly by taking the time derivative of (A9), using (A3), (A4), (A10)–(A12), and making use of the same identities and extension of sums to *K*:

(A22) $$\begin{array}{ccc} \frac{d}{dt}\Sigma & = & \sum_{i=1}^{K-1}\left({\left(x_{i}-\mu \right)}^{2}-{\left(x_{K}-\mu \right)}^{2}\right)\frac{dp_{i}}{dt} \end{array}$$

(A23) $$\begin{array}{ccc} & = & \sum_{i=1}^{K-1}\left({\left(x_{i}-\mu \right)}^{2}-{\left(x_{K}-\mu \right)}^{2}\right)\sum_{j=1}^{K-1}g_{ij}^{-1}\frac{\partial F}{\partial p_{j}} \end{array}$$

(A24) $$\begin{array}{ccc} & = & \sum_{i=1}^{K-1}p_{i}\left({\left(x_{i}-\mu \right)}^{2}-{\left(x_{K}-\mu \right)}^{2}\right)\left(\frac{\partial \mu^{⊤}}{\partial p_{i}}\nabla_{\mu }F+\frac{\partial \Sigma^{⊤}}{\partial p_{i}}\nabla_{\Sigma }F\right)\\ & & -\sum_{i=1}^{K-1}p_{i}\left({\left(x_{i}-\mu \right)}^{2}-{\left(x_{K}-\mu \right)}^{2}\right)\sum_{j=1}^{K-1}p_{j}\left(\frac{\partial \mu^{⊤}}{\partial p_{j}}\nabla_{\mu }F+\frac{\partial \Sigma^{⊤}}{\partial p_{j}}\nabla_{\Sigma }F\right) \end{array}$$

(A25) $$\begin{array}{ccc} & = & \sum_{i=1}^{K-1}p_{i}\left({\left(x_{i}-\mu \right)}^{2}-{\left(x_{K}-\mu \right)}^{2}\right)\left({\left(x_{i}-x_{K}\right)}^{⊤}\nabla_{\mu }F+{\left({\left(x_{i}-\mu \right)}^{2}-{\left(x_{K}-\mu \right)}^{2}\right)}^{⊤}\nabla_{\Sigma }F\right) \end{array}$$

(A26) $$\begin{array}{ccc} & & -\left(\Sigma -{\left(x_{K}-\mu \right)}^{2}\right)\sum_{j=1}^{K-1}p_{j}\left({\left(x_{j}-x_{K}\right)}^{⊤}\nabla_{\mu }F+{\left({\left(x_{j}-\mu \right)}^{2}-{\left(x_{K}-\mu \right)}^{2}\right)}^{⊤}\nabla_{\Sigma }F\right) \end{array}$$

(A27) $$\begin{array}{ccc} & = & \left(\langle {\left(x_{i}-\mu \right)}^{3}\rangle -\Sigma \left(x_{K}-\mu \right)+{\left(x_{K}-\mu \right)}^{3}\right)\nabla_{\mu }F \end{array}$$

(A28) $$\begin{array}{ccc} & & +\left(\langle {\left(x_{i}-\mu \right)}^{4}\rangle -2\Sigma {\left(x_{K}-\mu \right)}^{2}+{\left(x_{K}-\mu \right)}^{4}\right)\nabla_{\Sigma }F \end{array}$$

(A29) $$\begin{array}{ccc} & & -\left(\Sigma -{\left(x_{K}-\mu \right)}^{2}\right)\left(\left(\mu -x_{K}\right)\nabla_{\mu }F+\left(\Sigma -{\left(x_{K}-\mu \right)}^{2}\right)\nabla_{\Sigma }F\right) \end{array}$$

(A30) $$\begin{array}{ccc} & = & \left(\langle {\left(x_{i}-\mu \right)}^{3}\rangle -\Sigma \left(x_{K}-\mu \right)+{\left(x_{K}-\mu \right)}^{3}+\Sigma \left(x_{K}-\mu \right)-{\left(x_{K}-\mu \right)}^{3}\right)\nabla_{\mu }F \end{array}$$

(A31) $$\begin{array}{ccc} & & +\left(\langle {\left(x_{i}-\mu \right)}^{4}\rangle -2\Sigma {\left(x_{K}-\mu \right)}^{2}+{\left(x_{K}-\mu \right)}^{4}-\Sigma^{2}+2\Sigma {\left(x_{K}-\mu \right)}^{2}-{\left(x_{K}-\mu \right)}^{4}\right)\nabla_{\Sigma }F \end{array}$$

(A32) $$\begin{array}{ccc} & = & \langle {\left(x_{i}-\mu \right)}^{3}\rangle \nabla_{\mu }F+\left(\langle {\left(x_{i}-\mu \right)}^{4}\rangle -\Sigma^{2}\right)\nabla_{\Sigma }F \end{array}$$

(A33) $$\begin{array}{ccc} & = & 2\Sigma^{2}\nabla_{\Sigma }F \end{array}$$

In the final step we use the identity that the fourth central moment $\langle {\left(x_{i}-\mu \right)}^{4}\rangle =3\Sigma^{2}$ which is valid for the normal distribution.

## Appendix B. Selection and Newton’s Method

The integrated dynamics of the frequencies are

$$p_{i}\left(t\right)=p_{i}\left(0\right)\frac{1}{Z_{t}}e^{tf(x_{i})},$$

with normalization $Z_{t}=\sum_{i}p_{i}\left(0\right)e^{tf(x_{i})}$. The change in mean phenotype is proportional to the covariance between phenotype and fitness, (Price’s theorem)

$$\begin{array}{cc} \mu_{t}-\mu_{0} & =\frac{1}{Z_{t}}\sum_{i=1}^{K}p_{i}\left(0\right)\left(x_{i}e^{tf(x_{i})}-\mu_{0}Z_{t}\right). \end{array}$$

A taylor expansion of f(x) around $\mu_{0}$ to second order is

$$f\left(x\right)\approx f\left(\mu_{0}\right)+\left(x-\mu_{0}\right)\nabla f-\frac{1}{2}{\left(x-\mu_{0}\right)}^{2}\cdot C,$$

where ∇f is the vector of partial derivates of fitness evaluated at $\mu_{0}$, and C is the curvature, that is the negative of the matrix of second partial derivatives evaluated at $\mu_{0}$, and we use a shorthand for the outer product, e.g, $b^{2}=bb^{⊤}$, and $b^{2}\cdot A=b^{⊤}Ab$, for an arbitrary matrix *A*. The expansion is more conveniently written as

$$f\left(x\right)\approx F^{*}-\frac{1}{2}{\left(x-x^{*}\right)}^{2}\cdot C$$

where $F^{*}$ and $x^{*}$ are the approximate optimum fitness and phenotype respectively, and $C(x^{*}-\mu_{0})=\nabla f$.

If phenotypes are initially normally distributed then they remain normal after selection with the quadratic approximation of the fitness landscape, as can be seen from the integrated replicator dynamics. The resulting mean and inverse covariance are

$$\begin{array}{cc} \mu_{t}\; & =\Sigma_{t}\left(\Sigma_{0}^{-1}\mu_{0}+tCx^{*}\right)\\ \Sigma_{t}^{-1}\; & =\Sigma_{0}^{-1}+tC \end{array}$$

The change in the mean is

$$\begin{array}{cc} \mu_{t}-\mu_{0}\; & =\Sigma_{t}t\nabla f\\ \; & ={\left(\frac{1}{t}\Sigma_{0}^{-1}+C\right)}^{-1}\nabla f. \end{array}$$

Under strong selection in a quadratic fitness landscape the covariance shrinks to zero at a rate

$$\Sigma_{t}\to \frac{C^{-1}}{t}$$

## Appendix C. Genetic Load

Fitness is distributed as a noncentral chi-squared distribution, since fitness is a sum of squared values that are normally distributed. The difference between the optimum and the mean fitness:

(A34) $$\begin{array}{cc} F^{*}-F & =\frac{1}{2}\sum_{i}p_{i}{\left(x_{i}-x^{*}\right)}^{2}\cdot C\;\\ \; & =\frac{1}{2}\left(\Sigma_{t}+{\left(\mu_{t}-x^{*}\right)}^{2}\right)\cdot C \end{array}$$

which decomposes into an effective degrees of freedom $Tr(\Sigma_{t}C)$, and a noncentrality parameter ${\left(\mu_{t}-x^{*}\right)}^{2}\cdot C$. For strong selection this fitness difference aproaches zero as

$$F^{*}-F\to \frac{D}{2t}+O\left(t^{-2}\right)$$

where *D* is the dimension of *x*.

## Appendix D. Mutations

If mutations are normally distributed around each focal phenotype, the total covariance of all mutants will be the phenotype covariance plus the mutational covariance $\Sigma +\Sigma_{m}$. The associated mutational load is $\mathrm{Tr}(\Sigma_{m}C)$, and if mutations are standard normal, then the mutational load is the sum of the eigenvalues of C, or roughly the number of dimensions where fitness is sharply curved and not flat.

## Appendix E. Gradient Step in a Strong Selection Weak Mutation Model

In strong selection weak mutation dynamics of the Wright–Fisher model [2], a population is monomorphic (only one type), and changes type x by mutation-fixation events. A mutant has a fitness difference $f\left(x^{\prime }\right)-f\left(x\right)\approx \Delta x\nabla f\equiv s$, and the probability of fixation is

$$P_{\mathrm{fix}}\left(x^{\prime }|x\right)=\left\{\begin{array}{cc} 2s & s>0\\ 0 & s\leq 0 \end{array}\right..$$

The expected change in x is

$$E\left(\Delta x\right)=\int \Delta xP_{\mathrm{fix}}\left(x^{\prime }|x\right)P_{m}\left(x^{\prime }|x\right)dx^{\prime },$$

where $P_{m}\left(x^{\prime }|x\right)$ is the mutation distribution around *x*. For a normal mutation distribution $P_{m}\left(x^{\prime }|x\right)=N\left(x,\Sigma_{m}\right)$,

$$E\left(\Delta x\right)=\Sigma_{m}\nabla f$$
